# Integrating Graph Convolution and Attention Mechanism for Kinase Inhibition Prediction

**DOI:** 10.3390/molecules30132871

**Published:** 2025-07-06

**Authors:** Hamza Zahid, Kil To Chong, Hilal Tayara

**Affiliations:** 1 Department of Electronics and Information Engineering, Jeonbuk National University, Jeonju 54896, Jeollabuk-do, Republic of Korea; 202259032@jbnu.ac.kr; 2 Advances Electronics and Information Research Centre, Jeonbuk National University, Jeonju 54896, Jeollabuk-do, Republic of Korea; 3 School of International Engineering and Science, Jeonbuk National University, Jeonju 54896, Jeollabuk-do, Republic of Korea

**Keywords:** inhibition prediction, kinase inhibition prediction, graph neural network, graph convolution network, graph attention network, drug discovery

## Abstract

Kinase is an enzyme responsible for cell signaling and other complex processes. Mutations or changes in kinase can cause cancer and other diseases in humans, including leukemia, neuroblastomas, glioblastomas, and more. Considering these concerns, inhibiting overexpressed or dysregulated kinases through small drug molecules is very important. In the past, many machine learning and deep learning approaches have been used to inhibit unregulated kinase enzymes. In this work, we employ a Graph Neural Network (GNN) to predict the inhibition activities of kinases. A separate Graph Convolution Network (GCN) and combined Graph Convolution and Graph Attention Network (GCN_GAT) are developed and trained on two large datasets (Kinase Datasets 1 and 2) consisting of small drug molecules against the targeted kinase using 10-fold cross-validation. Furthermore, a wide range of molecules are used as independent datasets on which the performance of the models is evaluated. On both independent kinase datasets, our model combining GCN and GAT provides the best evaluation and outperforms previous models in terms of accuracy, Matthews Correlation Coefficient (MCC), sensitivity, specificity, and precision. On the independent Kinase Dataset 1, the values of accuracy, MCC, sensitivity, specificity, and precision are 0.96, 0.89, 0.90, 0.98, and 0.91, respectively. Similarly, the performance of our model combining GCN and GAT on the independent Kinase Dataset 2 is 0.97, 0.90, 0.91, 0.99, and 0.92 in terms of accuracy, MCC, sensitivity, specificity, and precision, respectively.

## 1. Introduction

Kinases are a special enzyme family that catalyzes the transfer of the phosphate group from Adenosine Triphosphate (ATP) to other proteins [1]. The transfer of the phosphate group from ATP to other proteins is known as phosphorylation, which is a complex mechanism involved in maintaining and regulating different cellular functions such as proliferation, cytoskeleton arrangements, nervous system function, apoptosis growth, etc. There are a total of 518 protein kinase genes in the human genome, comprising 1.7 percent of the human genome. Among these 518 protein kinase genes, 478 belong to the classical protein kinase family and the remaining 40 to atypical protein kinases [2]. Any abnormality or dysregulation in kinase activity is highly problematic and has consequences for diseases including diabetes, inflammatory diseases, cancer, and nervous disorders. Disordered kinases may be due to causes such as mutation, abnormal phosphorylation, chromosomal translocation, and epigenetic regulation [3].

Kinases are considered to be excellent drug targets because abnormalities are the reason for many diseases. To deal with abnormalities caused by kinases, it is very important to study drug molecules that can inhibit their activities. To date, many kinase inhibitors have been studied and found to be successful in oncologic therapy. However, many patients show high resistance to these drug molecules. In 2001, the U.S. Food and Drug Administration (FDA) approved a tyrosine kinase inhibitor called imatinib for oncologic therapy [4]. This approval marked the beginning of kinase inhibitors as the pivotal drug class in the field of oncology and beyond. According to the data from 2021, the US FDA has approved nearly 68 kinase inhibitors that are available to target the various protein kinases [5]. As the human kinome is composed of 518 protein kinases, a great deal of work and research is underway to study these as-yet understudied kinases. Investigating the chemical space of drug molecules targeting kinase proteins will deepen the understanding of kinase functions and facilitate the identification and optimization of specific kinase inhibitors.

In the past, many conventional approaches to drug design have been proven; however, these methods are characterized by being time-consuming, expensive, and laborious. The entire process of drug design from lead optimization to clinical trials spans approximately 12 years, and costs around USD 1.2 billion [6,7]. In contrast to these traditional techniques, many in silico approaches influenced by deep learning and machine learning frameworks are now being utilized, as they are cheaper and less time-consuming [8,9]. Similar approaches are being developed to forecast the inhibition activity of the kinase protein in the fields of drug design and bioinformatics [10].

Ref. [11] established the multitask deep neural network known as MTDNN, a classification model used for predicting the interacting profiles of kinase inhibitors against a panel of 391 kinases. They obtained a high auROC of 0.90 on the test data. Ref. [12] employed multitask machine learning for classifying highly and weakly potent kinase inhibitors. They employed Multi-Task (MT) machine learning models to predict the active compounds for the multiple targets, outperforming the conventional Single-Task (ST) learning. Ref. [13] developed machine learning as well as Multi-Task Deep Learning Methods (MTDNN) for activity prediction of kinase. They used extensive data from different databases comprising over 650,000 aggregated bioactivity annotations for more than 300,000 small molecules and consisting of 342 kinase targets. Their MTDNN model outperformed all the classical ST methods. Ref. [5] gathered an extensive dataset from different databases. They developed a multilayer perceptron model that predicts drug molecules as inhibitors or non-inhibitors for the kinase enzyme. One of their datasets yielded the optimal outcomes regarding accuracy, specificity, and sensitivity. Ref. [14] developed a web application that predicts the kinome-wide polypharmacology effect of small molecules based on their chemical structures. They deployed an MTDNN that was trained on more than 140,000 bioactivity data points for 391 kinases. Ref. [2] experimentally developed CancerOmicsNet, which consists of multimodal heterogeneous data for utilization with graph-based techniques. Their model was able to effectively learn the graphs and provided the best scoring methods for evaluating and ranking relevant kinases. Recent studies have shown GNNs’ remarkable versatility in drug discovery. The methodology of [15,16] highlights the use of geometric and self-supervised GNNs for molecular generation, virtual screening, and property prediction, providing improved interpretability and uncertainty control. Ref. [17] demonstrated that transfer learning with GNNs improves oral bioavailability prediction, while recent GNN-based DDI models employ attention mechanisms and substructure analysis to reveal key interaction features. Furthermore, [18] proposed a Drug Target–Disease Graph Neural Network (DTD-GNN) that models ternary relationships between drugs, targets, and diseases to identify drug repurposing candidates with superior predictive performance. These findings demonstrate that GNNs such as GCN, GAT, and their hybrid variants are highly effective in capturing molecular structure and feature relevance, making them well suited for drug discovery tasks such as kinase inhibition prediction.

A review of the literature reveals that the multi-layer perceptron model proposed by [5] provides the best result on data of similar size and type as used in our study. In their study, they divided the datasets into two parts based on the bioactivities of the molecules. Compounds with bioactivity values of 10 micromoles (μM) or less were classified as active and included in Dataset 1. Similarly, compounds having bioactivity values of 1 micromole or less were classified as active and included in Dataset 2. Dataset 1 comprises 796,495 compounds that target 406 kinases, encompassing a total of 6,749,276 bioactivity annotations. In contrast, Dataset 2 contains 795,669 compounds targeting 363 kinases with a total of 6,723,637 bioactivity annotations.

**Figure 1:**
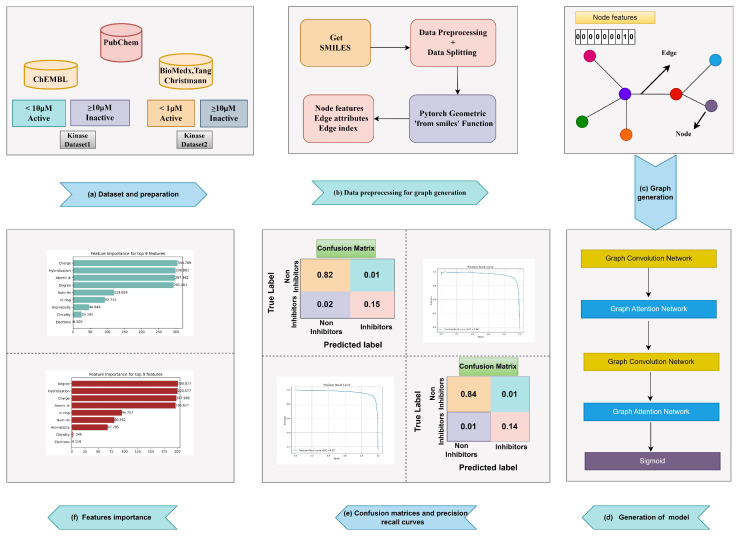
Workflow of the study: (**a**) the repositories from which the datasets were extracted based on the bioactivities of the molecules, (**b**,**c**) the preprocessing, data splitting, and process of graph generation using the Pytorch Geometric library with SMILES data, (**d**) model architecture based on graph convolutional and graph attention networks for feature extraction, (**e**) confusion matrices and precision–recall curves based on actual and predicted outputs, (**f**) range of important node features for the model output prediction.

In this article, we introduce GNN-based models for predicting the inhibition activities of kinase proteins. Data are taken from different databases. Based on the bioactivities of the small molecules, the data are divided into two parts, i.e., Datasets 1 and 2. These datasets are represented using the Simplified Molecular Input Line Entry System (SMILES) format, then transformed into graphs using the Pytorch Geometric library. Then, two layers of separate graph convolutional networks are used, each followed by a graph attention network inherited from the Pytorch Geometric library [19]. These networks are trained on two different datasets based on the bioactivities of the molecules. Different evaluations such as balanced accuracy, sensitivity, specificity, and Mathews correlation coefficient are calculated, providing a much better assessment than previous models used for kinase inhibition prediction. The results are compared with those of the previous study by [5], which achieved the best outcomes when training on large datasets followed by subsequent testing on independent datasets. The workflow of our study can be seen in Figure 1.

## 2. Results and Discussion

### 2.1. Cross-Validation Performance of the Proposed Model

For both kinase datasets, we used 90% of the data for training the separate GCN and GAT models. The technique of early stopping with a patience value of 20 was introduced to monitor the training and validation losses. This technique halts the training process if the validation loss does not decrease for 20 consecutive epochs, thereby preventing the model from overfitting [20,21]. The loss curves of the training and validation data can be seen in Figures S1 and S2 in the Supplementary Information File S1. For each of the datasets, different classification metrics such as accuracy, balanced accuracy, MCC, sensitivity, and specificity were measured. The mean values of accuracy, balanced accuracy, MCC, sensitivity, and specificity on all ten folds for Dataset 1 were 0.97, 0.94, 0.89, 0.90, and 0.98, respectively. Similarly, the mean values of accuracy, balanced accuracy, MCC, sensitivity, and specificity on the ten folds for Dataset 2 were 0.97, 0.95, 0.90, 0.91, and 0.98, respectively. The details of these performance metrics for both datasets of kinase can be seen in Table 1.

### 2.2. Model Performance on the Independent Dataset

**Figure 2:**
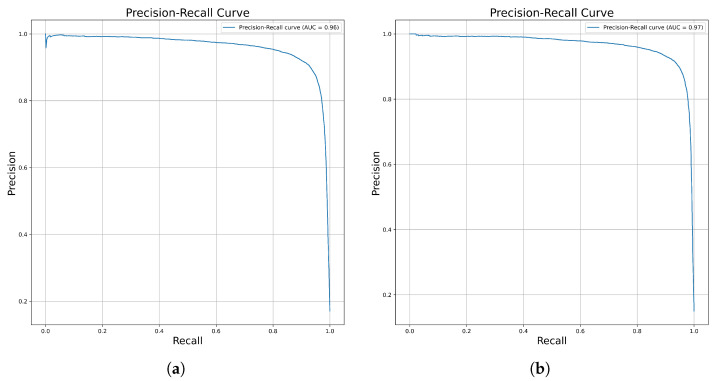
Precision–Recall (PR) curves demonstrating the classification performance of the model on Kinase Dataset 1 (**a**) and Kinase Dataset 2 (**b**), highlighting the tradeoff between precision and recall across different threshold values.

The 10% of the data left out of the total dataset (i.e., both of the kinase datasets) served as independent data for evaluating the performance of the model. The number of independent samples on which the prediction was performed was 76,959 for Kinase Dataset 1 and 75,460 for Kinase Dataset 2. An independent dataset or holdout for both datasets was included in the SMILE representation and subsequently transformed into a graph through the Pytorch Geometric library. The saved training models were loaded, then predictions were performed using these independent datasets. The confusion metrics for both of the datasets were constructed, from which false positive, false negative, true positive, and true negative values are calculated, as shown in Figures S3 and S4 in the Supplementary Information File S1. The mean values of accuracy, balanced accuracy, MCC, sensitivity, specificity, etc., on both kinase datasets were measured, as shown in Table 1. Furthermore, because of data imbalance, the precision and recall curves for both kinase datasets were plotted, which can be seen in Figure 2.

### 2.3. Experimental Validation of Kinase Inhibitors Using Deep Learning and Molecular Docking

To experimentally validate the predictions of our model, we first identified a set of FDA-approved drugs from the literature that are known to act against various kinases [22]. The selected compounds (Tovorafenib, Fostamatinib, Abrocitinib, and Binimetinib) were evaluated against cancer-associated kinase targets, including B-Raf/C-Raf proto-oncogene serine/threonine-protein kinases (B-/C-Raf), spleen tyrosine kinase (Syk), Janus kinase 1 (JAK1), and mitogen-activated protein kinase kinase (MEK1), respectively. Initially, these drugs were screened using our predictive model, generating probability scores indicating their potential as kinase inhibitors. The results can be seen in File S2 of the Supplementary Information.

**Figure 3:**
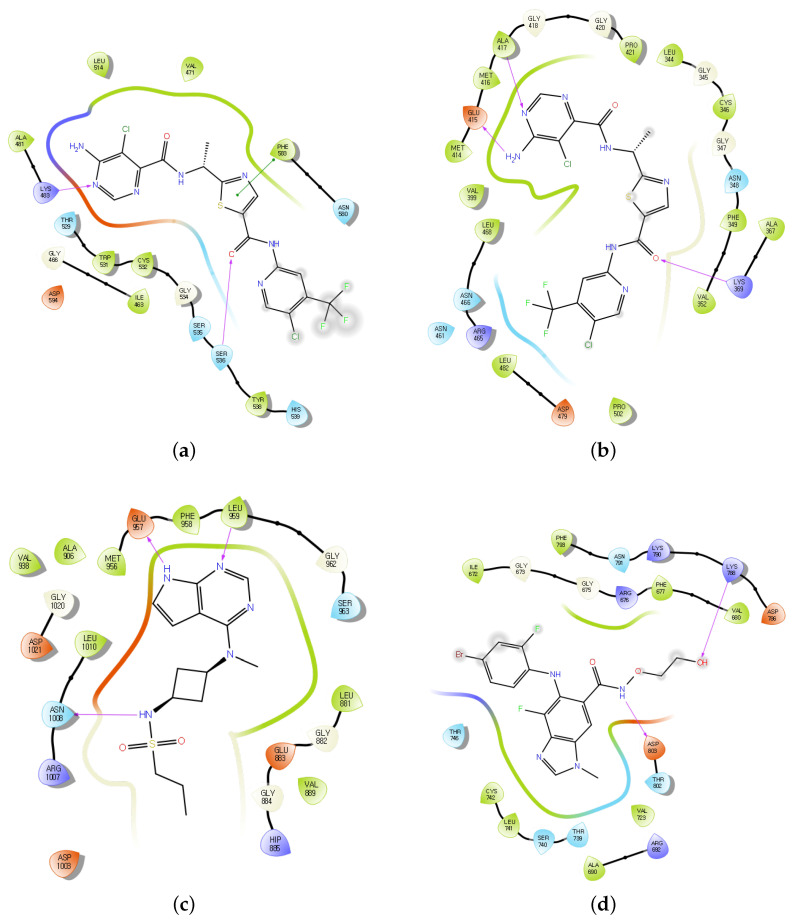
Docking poses of selected FDA-approved drugs with their respective kinase targets: (**a**) Tovorafenib–B-/C-Raf, (**b**) Fostamatinib–Syk, (**c**) Abrocitinib–JAK1, and (**d**) Binimetinib–MEK1.

To further validate these predictions, molecular docking was performed using Maestro (Schrodinger 2023-2 suites) [23]. The crystal structures of the target kinases were retrieved from Protein Data Bank (https://www.rcsb.org/) and prepared using the Protein Preparation Wizard in Maestro (Schrodinger 2023-2 suites. The small molecules were processed using LigPrep in Maestro (Schrodinger 2023-2 suites), and binding pockets were identified using PURESNET 2.0 [24]. Docking simulations were then carried out using the GLIDE docking protocol. The performance of each drug–target interaction was evaluated using GLIDE GSCORE which estimates binding affinity. The resulting scores ranged from −6.101 kcal/mol to −9.591 kcal/mol, suggesting favorable binding interactions between the drugs and the selected kinases. These results support the effectiveness of the proposed model and the selected compounds as potential kinase inhibitors. The interactions between the molecules and their target kinases are depicted in Figure 3.

### 2.4. Comparison with Established Models

**Figure 4:**
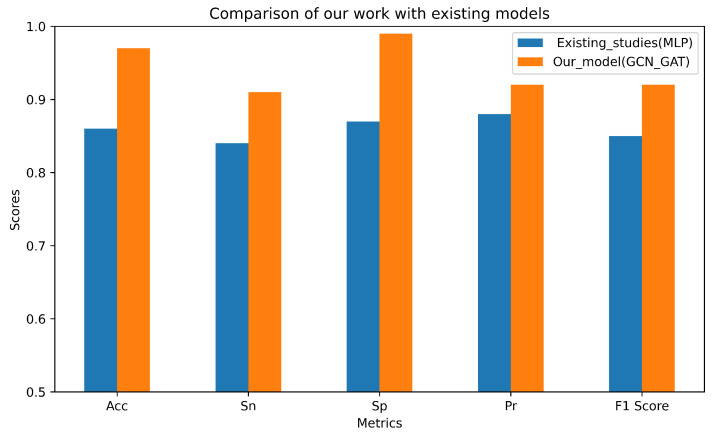
Bar chart highlighting the differences between our study results and those from the previous study.

To evaluate the robustness and reliability of our models, they were benchmarked against established studies. Utilizing similar data to [5], we compared our results with their best outcomes on Dataset 2. Upon comparison, it was found that our GCN–GAT model outperformed the MLP model from [5] on the independent dataset in terms of accuracy, specificity, sensitivity, precision, and F1-score. A comparison of results between our GCN–GAT model and their MLP model is shown in Figure 4.

The strategic combination of GCNs and GATs in our model plays a critical role in enhancing prediction performance for kinase inhibition. GCN layers effectively capture the global topological structure of molecular graphs by aggregating information from neighboring nodes; however, they treat all neighbors equally, which can result in a loss of important local information. On the other hand, GAT layers incorporate an attention mechanism that assigns learned importance weights to each neighboring node, allowing the model to focus on substructures that are more relevant to molecular activity. By integrating both GCN and GAT layers, the combined GCN–GAT model benefits from global structural awareness and localized attention, producing richer molecular representations. This combined architecture contributes to the improved predictive capability observed when compared to both the standalone GCN and the previously established MLP baseline.

### 2.5. Graph Explainability and Feature Importance

**Figure 5:**
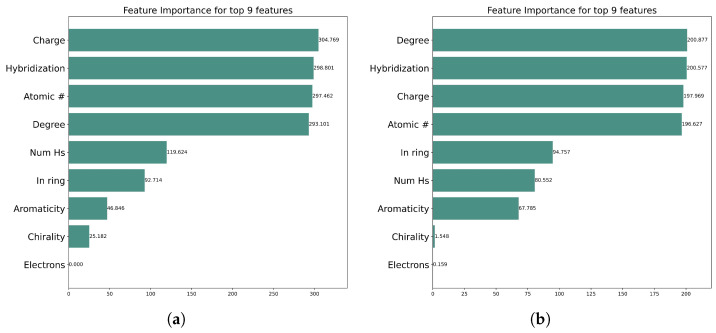
Feature importance for Kinase Datasets 1 (**a**) and 2 (**b**) as identified by GNNExplainer, highlighting the key features driving model predictions.

Graph Neural Networks (GNNs) are considered to be the most powerful tool for dealing with graph data. GNNs combine nodes and edge information to create embeddings that are used for various prediction purposes. While incorporating the graph structure and node features, the models become more and more complex. Because of this complexity, it is not easy to explain the prediction made by GNNs [25]. In this study, we employ GNNExplainer, which is an approach that explains the prediction made by the GNNs. GNNExplainer provides a subgraph along with those node features that are most important for making predictions [26]. The important node features for both Kinase Dataset 1 and Kinase Dataset 2 that contribute the most to the model’s predictions are shown in Figure 5. Figure 5a illustrates that for Kinase Dataset 1 the most important node features for model output prediction are the charge, hybridization, and atomic number. Similarly, Figure 5b shows that the degree, hybridization, and charge are the node features that contribute most significantly to the model’s output prediction for Kinase Dataset 2.

In our model, the charge, degree, hybridization, and atomic number are the most significant features influencing the prediction for both datasets. These features are not only statistically important in model predictions but also chemically and biologically relevant in the context of kinase–inhibitor interactions. The charge plays a crucial role in determining electrostatic interactions between inhibitor molecules and residues within the kinase binding pocket, directly impacting binding affinity. Hybridization states affect the spatial orientation and geometry of atoms, influencing how well a molecule fits into the active site. The atomic number reflects the elemental identity of atoms, which determines their ability to participate in hydrogen bonding or metal coordination, both of which are common in kinase–inhibitor binding. Additionally, the degree of an atom relates to its local connectivity and steric environment, which can influence accessibility or flexibility at the binding interface. The consistent appearance of these features across both datasets suggests that the model is not only learning from the data but also aligning with known principles of medicinal chemistry.

## 3. Materials and Methods

### 3.1. Data Collection and Preparation

The data were collected from different databases, including ChEMBL [27], PubChem [28], PKIS set [29], Tang set [30], BioMedx set [31], and Christmann2016 [32]. A similar dataset was previously used by [5] in their work. The preprocessing steps followed by [5] are summarized as follows: (1) the compound structures were standardized using the RDKit (2022.09.5) and KNIME tools (version 2.0) [33,34]; (2) all compounds listed as mixtures were separated, and the largest compounds were considered; (3) all compounds having collisions or unconventional stereos were removed, with only organic compounds retained; (4) duplicates were checked and removed. The remaining compounds were classified as active and inactive based on their bioactivities (IC50/ki/kd). Two datasets were curated based on the bioactivity values of the compounds. Compounds with bioactivity values of 10 μM or less were classified as active and included in Dataset 1. Compounds having bioactivity values of 1 μM or less were classified as active and included in Dataset 2. Dataset 1 comprised 769,593 compounds targeting 406 kinases, encompassing a total of 6,749,276 bioactivity annotations. In contrast, Dataset 2 contained 754,608 compounds targeting 363 kinases, with a total of 6,723,637 bioactivity annotations. For both datasets, the data were divided into two parts. The first part contained 90% of the data, which acted as the training data, while the remaining 10% was held out. After preprocessing, Kinase Dataset 1 included 692,633 compounds. These were used in a 10-fold cross-validation framework, where nine folds were dedicated to training and the remaining fold to validation [35]. The held-out 10% of the total data served as independent data for evaluating the model’s performance. After preprocessing Dataset 2, we retained 754,608 samples. Of these, 679,147 samples were utilized for training using 10-fold cross-validation, in which nine folds were employed for training and the remaining fold for validation. The remaining 75,460 samples served as holdout data for evaluating the model’s performance. The data for both datasets are available in SMILES format, which consists of strings that concisely represent the chemical structure [36,37]. These SMILES data were converted into graphs which act as input for the GNN.

### 3.2. Model Evaluation Measures

Accuracy, sensitivity (true positive rate), specificity (true negative rate), and ROC–AUC are among the measures used for classification tasks [38]. However, the balanced accuracy (Ba) and Matthews correlation coefficient (MCC) are mostly used when dealing with imbalanced data [39]. The evaluation measures along with their mathematical formulas are represented in the following equations:

(1) $$TPR=Sn=Recall=\frac{TP}{FN+TP}$$

(2) $$TNR=Sp=\frac{TN}{FP+TN}$$

(3) $$Ba=\frac{TPR+TNR}{2}$$

(4) $$Precision=\frac{TP}{FP+TP}$$

(5) $$F1\_Score=2\times \frac{Precision\times Recall}{Precision+Recall}$$

(6) $$MCC=\frac{TP\times TN-FN\times FP}{\sqrt{\left(TP+FN)\times (TP+FP)\times (TN+FN)\times (TN+FP\right)}}$$

where FP, FN, TN, and TP represent false positive, false negative, true negative, and true positive, respectively.

### 3.3. Graph Generation and Feature Extraction

After data preprocessing, the SMILES data were passed to Pytorch Geometric, which is a library used for a wide range of applications related to structured data [19]. The “from SMILES” function of the Pytorch Geometric library was used to convert the SMILES data into the essential components of the graph: node features, edge indices, and edge attributes. In this study, we focus only on the node features, using a total of nine node features: atomic number, chirality, degree, formal charge, num_hs, num_radical_electrons, hybridization, is_aromatic, and is_in_ring. These features all have different ranges and descriptions. The ranges and their descriptions are provided in Table 2.

### 3.4. Graph Based Models Selection

The choice or selection of models is always based on the type of data available. In this study, we deployed a GNN to learn the data. The graph simply consists of nodes, or vertices, and connections between these nodes, called edges. The information about these connections in the graph is represented by the adjacency matrix [40]. The connection between the nodes in the adjacency matrix is represented by 1 or 0, where 1 shows that the connection exists and 0 indicates that there is no connection. A GNN uses the neighbor information of all the neighboring nodes and provides a new output called an embedding [41]. These node embeddings store both the structural and feature information of other neighboring nodes in the graph, which indicates that these nodes know something about the other nodes. The purpose of these embeddings is to predict the output based on the type of task. These predictions can be at the node level, edge level, or graph level. We can aggregate or combine all of these node-level predictions in a certain way, such as mean, max, etc., to perform graph-level predictions. The core of GNN is the message-passing layer. Message passing is carried out by gathering the current node information from the nearby nodes and combining it in certain ways to obtain the new node embedding. Message-passing layers are involved in the aggregation and updates on different node embeddings. Based on these aggregate and update functions, there can be many variants of GNNs. The two GNN variants used in our work are GCN and GAT, which are explained in detail below.

#### 3.4.1. Graph Convolutional Networks

GCN was first proposed by Thomas Kipf and Max Welling in 2016. The main idea behind GCN is to apply the convolution over the graph instead of the 2D array [42]. GCN was designed for semi-supervised node classification to learn the node-level features in the graph. However, for our work on inhibition prediction of kinases, a graph representation of the drug molecules is required. There are a large number of techniques that try to aggregate or combine information from the learned node features to produce the graph-level representation, including average, sum, and max pooling [43,44]. In our problem, we use global max pooling for these purposes, as it provides better performance.

- Every graph convolution layer transforms the node features X by aggregating the information from other neighboring nodes and itself using the adjacency matrix. The expression of the GCN can be written as follows:
  (7) $$G=\sigma \left({\tilde{D}}^{-\frac{1}{2}}\tilde{A}{\tilde{D}}^{-\frac{1}{2}}X\Theta \right)$$
  where X represents the node features matrix, $\tilde{D}$ represents the degree of the adjacency matrix $\tilde{A}$ with self-loop added, Θ is the trainable weight matrix, σ is the activation function, $\tilde{A}=A+I$ represents the adjacency matrix with self-loop added, and I is the identity matrix.

#### 3.4.2. Graph Attention Networks

Like GCN, GAT is one of the most popular GNN architectures. It was first introduced by [45] in 2017 based on the simple idea that some nodes are more important than others. In contrast to GCNs, which utilize static weights, GATs employ self-attention mechanisms to dynamically assign weights to node features, allowing for more flexible and context-aware representations. The process of graph attention is defined as follows:

- Consider a set of nodes each having some input node features. These features are passed as input to the GAT layer. The set of features for all the input nodes can be represented as
  (8) $$g=\left\{\vec{g_{1}},\vec{g_{2}},\vec{g_{3}},\vec{g_{4}},\vec{g_{5}},\ldots ,\vec{g_{N}}\right\},\;\mathrm{where}\;\vec{g_{i}}\in R^{F},$$
  where $\vec{g_{i}}$ is the feature vector of node i with features F.
- The GAT layer produces a new set of node features as output, denoted as
  (9) $$g^{\prime }=\left\{\vec{g_{1}^{\prime }},\vec{g_{2}^{\prime }},\vec{g_{3}^{\prime }},\vec{g_{4}^{\prime }},\vec{g_{5}^{\prime }},\ldots ,\vec{g_{N}^{\prime }}\right\},\;\mathrm{where}\;\vec{g_{i}^{\prime }}\in R^{F^{\prime }},$$
  where $\vec{g_{i}^{\prime }}$ is the updated feature of node i with node features $F^{\prime }$.
- To transform the input features into higher-level features a learnable linear transformation using a shared matrix W is applied to every node, where $W\in R^{F^{\prime }\times F}.$ Next, a self-attention mechanism “*a*” is used to calculate the attention coefficient $e_{ij}$ which determines the importance of neighboring node *j*’s features to node *i*, after which the raw attention scores $e_{ij}$ are normalized using the softmax function as follows:
  (10) $$e_{ij}=a\left(W\vec{g_{i}},W\vec{g_{j}}\right)$$
  (11) $$\alpha_{ij}={\mathrm{softmax}}_{j}\left(e_{ij}\right)=\frac{\exp(e_{ij})}{\sum_{k\in N_{i}}\exp\left(e_{ik}\right)}$$
  where $N_{i}$ is the neighboring node of node *i*.
- For each neighbor *j*, the features of the nodes are first transformed using the weight matrix W. These transformed features are then multiplied by the normalized attention scores $\alpha_{ij}$. Summing these weighted scores and applying a nonlinearity σ yields new features for node *i*, as provided in the following equation:
  (12) $$\vec{g_{i}^{\prime }}=\sigma \left(\sum_{j\in N_{i}}\alpha_{ij}W\vec{g_{j}}\right).$$

### 3.5. Model Interpretation and Hyperparameters

**Figure 6:**
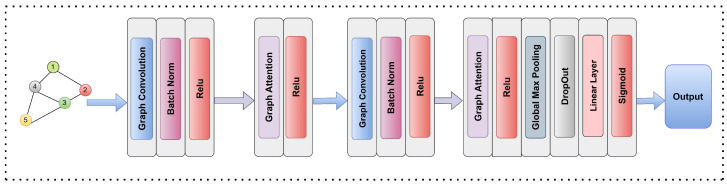
Illustration of the combined GCN–GAT architecture for predicting kinase inhibition. The architecture integrates multi-stage graph convolution and attention mechanisms, followed by global pooling and dense layers for final output generation.

First, a simple GCN architecture was developed by using the two layers of the GCN and training this architecture on the two kinase datasets; however, the results were not satisfactory when compared to the previous study. Thus, an architecture combining GCN and GAT was developed, as shown in Figure 6. The input data in the form of a graph consisting of the node features is allowed to pass to the GCN, followed by batch normalization and a relu function. The output from the relu function is passed to the graph attention, which utilizes the attention mechanism, then the output is passed again to a relu function. The same process is repeated once more, then a global max pooling layer is applied to shifts the node features by aggregating the node features to the graph level. The global max pooling layer is followed by a dropout layer, a linear layer, and a sigmoid function at the end because of the classification problem. The use of the graph attention layer with graph convolution has a significant impact on the model’s output prediction. The attention mechanism allows the network to weigh the importance of the neighboring node’s feature. This enables more relevant and context-based aggregation, leading to more discriminative node representation. By focusing on the most important neighboring nodes, the attention layers allow the network to reduce the impact of noisy or less relevant connections, enabling the network to arrive at more robust predictions and better generalizations.

When training deep learning or machine learning models, each model and dataset requires a set of different hyperparameters. The values of these hyperparameters can be adjusted by carrying out multiple experiments and measuring the best model performance. This process is known as hyperparameter tuning, and can be manual or automated using different methods. Regardless of which method is adopted, the goal is to track the results of the random experiments. The hyperparameters and their values are listed in Table 3.

## 4. Conclusions

Identifying hit compounds that can inhibit the activity of the enzyme or protein is still a challenging task. Nearly 90 percent of drug compounds fail to meet FDA standards to become drugs [46]. Research is focused on studying compounds that can target disease-causing proteins. Our study adopts a similar approach to predict the inhibition activity the kinase enzyme. Kinase is a crucial enzyme involved in regulating complex functions such as cell signaling. Mutations in kinase can cause serious diseases such as cancer, leukemia, neuroblastomas, glioblastomas, and more [47]. Looking at these concerns, we developed a graph neural network that predicts the inhibition activity of kinase. Two different graph neural networks, including a GCN and a combined GCN and GAT, were developed and trained on the two different datasets of kinases consisting of a wide range of small drug compounds using stratified-fold cross-validation techniques [48]. Our combined GCN and GAT model generalized well and provided the best results when tested on a wide range of independent datasets for both Kinase Dataset 1 and Kinase Dataset 2 in terms of accuracy, MCC, sensitivity, specificity, precision, and F1-score. On the independent Kinase Dataset 1, the values of accuracy, MCC, sensitivity, specificity, and precision were 0.96, 0.89, 0.90, 0.98, and 0.91, respectively. Similarly, the performance of our model combining GCN and GAT on the independent Kinase Dataset 2 in terms of accuracy, MCC, sensitivity, specificity, and precision was 0.97, 0.90, 0.91, 0.99, and 0.92, respectively. Based on this positive evaluation and robust generalization, our work can be extended to prediction of the inhibition activity of cytochrome p450 as well as potential neurotoxicity, cardiotoxicity, and hepatotoxicity, which helps to better understand adverse drug reactions and develop safer therapeutic agents.

## Figures and Tables

**Table 1:** Performance metrics demonstrating the comparison between 10-fold cross-validation and the holdout dataset for Kinase Datasets 1 and 2.

Datasets	Performance on the 10 Folds							Performance on Holdout Data						
	Acc	Ba	MCC	Sn	Sp	Pr	F1	Acc	Ba	MCC	Sn	Sp	Pr	F1
Dataset 1 (GCN_GAT)	0.97	0.94	0.89	0.90	0.98	0.92	0.91	0.96	0.94	0.89	0.90	0.98	0.91	0.90
Dataset 2 (GCN_GAT)	0.97	0.95	0.90	0.91	0.98	0.92	0.91	0.97	0.95	0.90	0.91	0.99	0.92	0.92
Dataset 1 (GCN)	0.92	0.84	0.73	0.80	0.97	0.83	0.77	0.92	0.84	0.72	0.71	0.97	0.83	0.76
Dataset 2 (GCN)	0.94	0.85	0.74	0.72	0.97	0.83	0.77	0.93	0.84	0.74	0.72	0.97	0.83	0.77

Note: Acc = accuracy, Ba = balanced accuracy, MCC = Matthews correlation coefficient, Sn = sensitivity, Sp = specificity, Pr = precision, F1 = F1-score.

**Table 2:** Summary of node features used in the molecular graph representation along with their respective ranges and brief descriptions.

Node Features	Range and Description
Hybridization	S, SP, SP3, etc.
Degree	00–11
Formal charge	−05–07
No. of Hs	00–09
No. of radical electrons	00–05
Atomic number	01–119
Is aromatic	Boolean
Is in ring	Boolean
Chirality	Atom chirality

**Table 3:** List of hyperparameters utilized in model training along with their specific values.

Hyperparameters	Values
Learning rate	0.0001
Batch size	32
GCN layers	2
GAT layers	2
Dropout rate	0.4
Pooling layer	global max
Activation function	Relu
Optimizer	Adam

## Data Availability

The training and test datasets for Kinase Dataset 1 and Kinase Dataset 2 used in our study are available on GitHub at https://github.com/HamzaZahidKhan0345/kinase-inhibition-prediction (accessed on 2 July 2025). The source code utilized in our study is also available on GitHub at https://github.com/HamzaZahidKhan0345/kinase-inhibition-prediction (accessed on 2 July 2025).

## Supplementary Materials

The following supporting information can be downloaded at: https://www.mdpi.com/article/10.3390/molecules30132871/s1, File S1—Figure S1: Comparison of training and validation loss curves on Kinase Dataset 1; Figure S2: Comparison of training and validation loss curves on Kinase Dataset 2; Figure S3: Confusion matrices for Kinase Dataset 1, illustrating the distribution of true positives, true negatives, false positives, and false negatives in the model’s predictions; Figure S4: Confusion matrices for Kinase Dataset 2, illustrating the distribution of true positives, true negatives, false positives, and false negatives in the model’s predictions; File S2: Experimental validation using deep learning and molecular docking.
