# Supplementary material for: Integrating Graph Convolution and Attention Mechanism for Kinase Inhibition Prediction
Source: Molecules. 2025 Jul 6;30(13):2871. doi: 10.3390/molecules30132871 (PMC12251378; doi:10.3390/molecules30132871)
Supplement: Supplementary file 1 [file molecules-30-02871-s001.zip › File S1 supplementary information.pdf]

Integrating Graph Convolution and Attention Mechanism for Kinase Inhibition Prediction

Hamza Zahid[1], Kil To Chong[1,2*] ,Hilal Tayara[3*]

1 Department of Electronics and Information Engineering, Jeonbuk National University, 54896, Jeonju, South Korea.

2 Advances Electronics and Information Research Centre, Jeonbuk National University, 54896, Jeonju, South Korea.

3 School of International Engineering and Science, Jeonbuk National University, 54896, Jeonju, South Korea.

* Correspondence: {kitchong@jbnu.ac.kr, hilaltayara@jbnu.ac.kr}

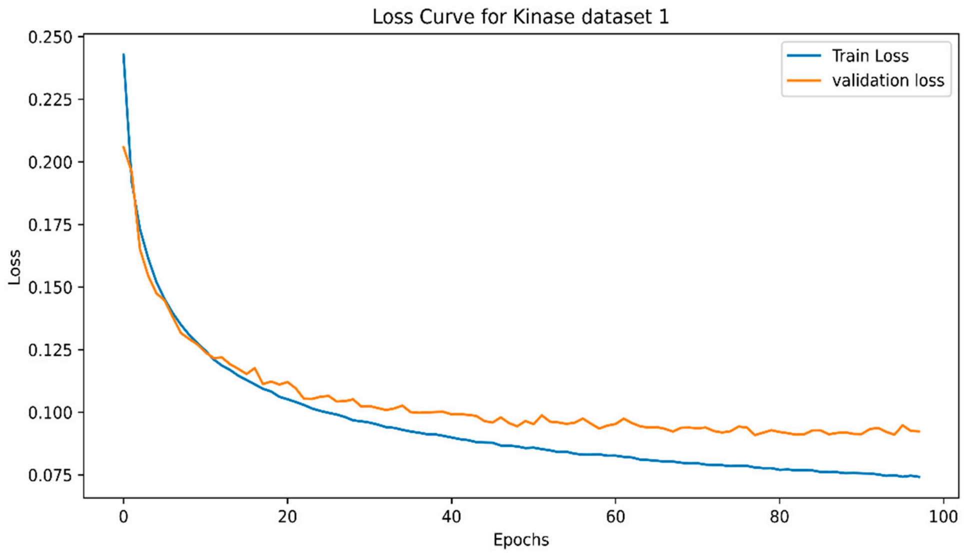

Figure S1: Comparison of training and validation loss curves on Kinase Dataset 1. The curves illustrate model learning behavior and convergence over training epochs.

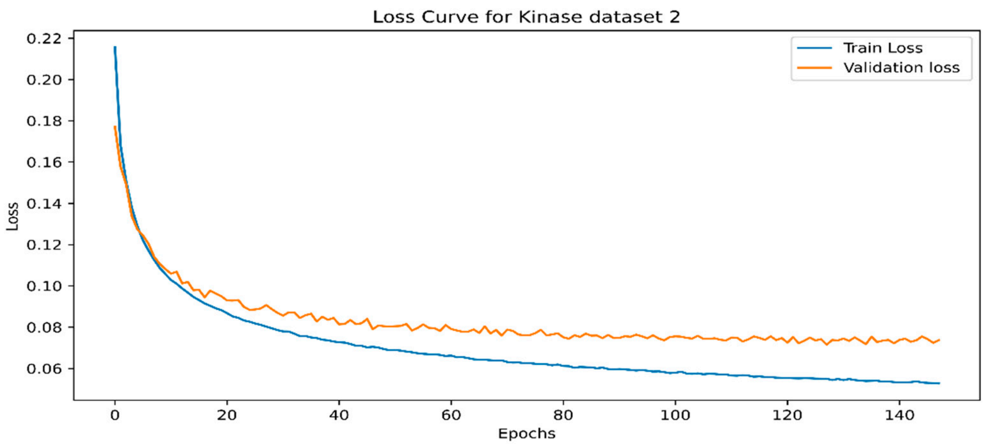

Figure S2: Comparison of training and validation loss curves on Kinase Dataset 2. The curves illustrate model learning behavior and convergence over training epochs.

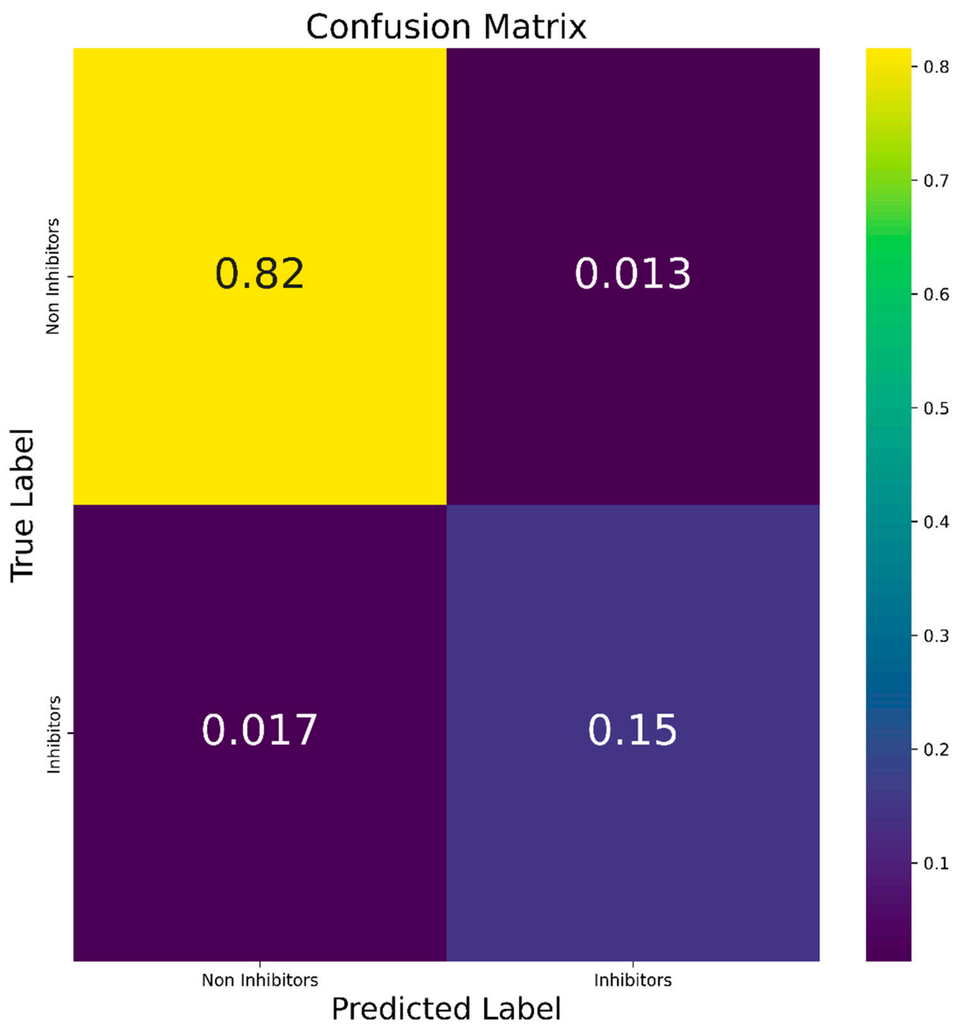

Figure S3: Confusion matrices for Kinase Dataset 1, illustrating the distribution of true positives, true negatives, false positives, and false negatives in the model's predictions.

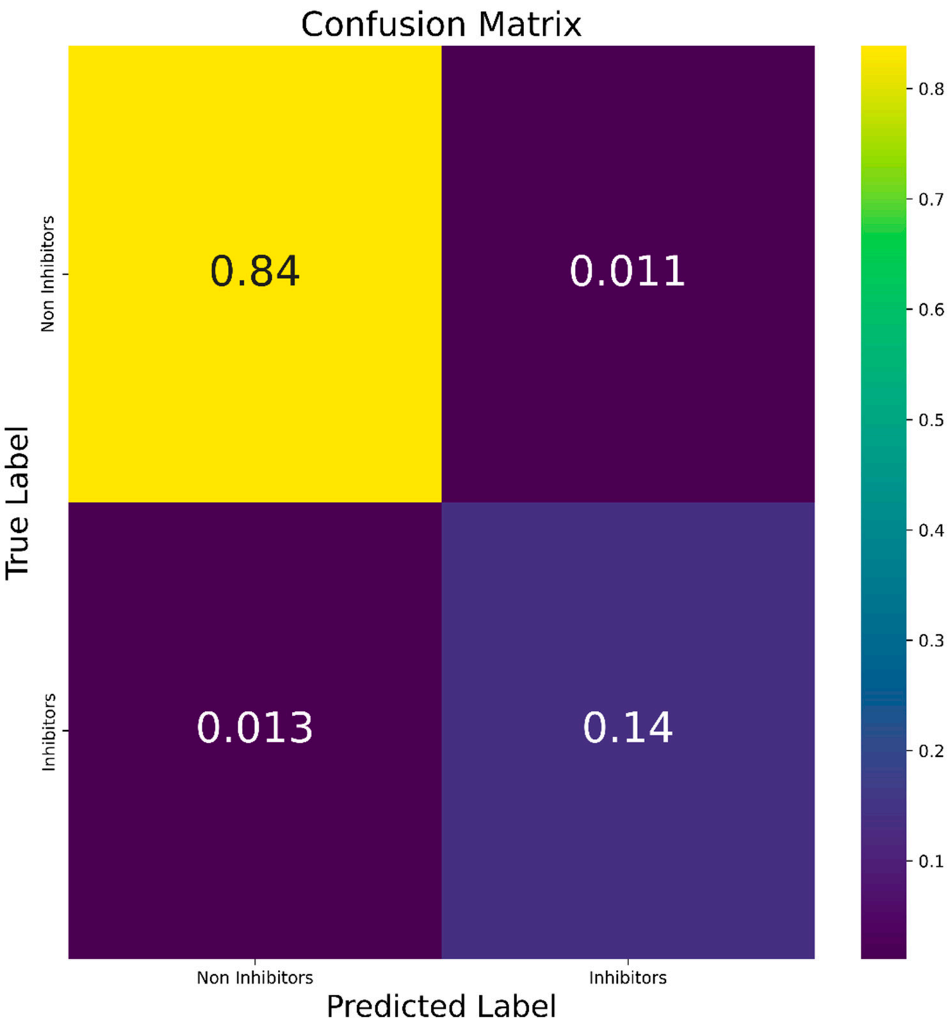

Figure S4: Confusion matrices for Kinase Dataset 2, illustrating the distribution of true positives, true negatives, false positives, and false negatives in the model's predictions.
